# Investigating the effect of Er,Cr:YSGG laser agitation of sodium hypochlorite on the removal of mature biofilm in the complex root canal systems using atomic force microscopy

**DOI:** 10.34172/joddd.2023.40463

**Published:** 2023-11-11

**Authors:** Ghufran Ismail Ibrahim, Hussein Ali Jawad

**Affiliations:** University of Baghdad, Institute of Laser for Postgraduate Studies, Baghdad, Iraq

**Keywords:** Atomic force microscope, Biofilm, Enterococcus faecalis, Er,Cr:YSGG laser, Passive ultrasonic activation, 5.25% Sodium hypochlorite

## Abstract

**Background.:**

Endodontic infections caused by remaining biofilm following disinfection with chemical fluids encourage secondary bacterial infection; hence, employing laser pulses to activate the fluids is advised to improve microbial biofilm clearance. This study investigated the performance of Er,Cr:YSGG laser in photon-induced photoacoustic streaming (PIPS) agitation of 5.25% sodium hypochlorite (NaOCl) to enhance the removal of mature Enterococcus faecalis (E. faecalis) biofilms in complex root canal systems.

**Methods.:**

The mesial roots of the lower first and second molars were separated and inoculated with E. faecalis bacteria for 30 days. The roots were irrigated with 5.25% NaOCl, some of them were agitated with passive ultrasonic irrigation (PUI), and the other roots were agitated by Er,Cr:YSGG laser using PIPS at 60 µs/pulse, 5 Hz, and 0.25, 0.5, 0.75, 1, and 1.25 W. An atomic force microscope (AFM) was used as a new method to obtain the results in the isthmus area; the results that have been obtained from each group were compared with each other. ANOVA was utilized to compare the means of the test groups.

**Results.:**

Based on the AFM and SEM analyses, laser agitation and passive ultrasonic activation groups have shown higher antimicrobial efficacy than the conventional syringe irrigation group (*P*<0.05).

**Conclusion.:**

Based on the findings of this investigation, the agitation of 5.25% NaOCl solution by Er,Cr:YSGG laser in PIPS at (60 µs/pulse, 5 Hz, 1.25 W) offers better mature bacterial biofilm removal in the mesial root of lower human molars than the same irrigant with syringe irrigation and passive ultrasonic activation technique.

## Introduction

 The structure of the root canal system, which consists of the isthmus, apical deltas, and accessory and lateral canals, is complicated and unexpected, making complete clearance of bacterial biofilms challenging.^1^ Isthmuses are typically present in the mesial roots of lower molars, where they may capture a significant amount of debris throughout the process of root canal preparation.^2^ The gram-positive facultative anaerobic bacterium *Enterococcus faecalis* is frequently detected in endodontic therapy failure.^3^ The most commonly used antibacterial fluid is sodium hypochlorite (NaOCl), a potent disinfectant with some properties, such as tissue dissolving and proteolytic effects on microbes, and is used at concentrations ranging from 0.5% to 6%.^4^ Erbium, chromium-doped yttrium, scandium, gallium, and garnet (Er,Cr:YSGG) is a type of water-absorbing laser with a wavelength of 2780 nm.^5,6^ Hydrokinetic energy for this laser has been suggested to promote dental canal disinfection with no thermal harm to the underlying tissue.^7^ Photon-induced photoacoustic streaming (PIPS) is a newly introduced laser technique to increase irrigation solution activity.^8^ Using ablative lasers in PIPS with a water-based solution creates cavitation, defined by the development of enormous oval vapor bubbles that expand and implode. This expansion generates high pressure that decreases as the bubbles rupture after 100‒200 µs, and re-entry of solution into the root canal produces the additional cavitation effects. As a result, the laser serves as a fluid pump. Atomic force microscopy (AFM) is a useful technique for studying the shape and texture of various surfaces, including roughness, waviness, and flaws.^9,10^ This method has been widely utilized to investigate the mechanisms of antibacterial substance activity on bacteria, and it acts as a complementary tool for the biologist.^11^ Kishen et al^12^ evaluated the effects of endodontic irrigation fluids on the adhesion of *E. faecalis* to root canal dentin by AFM and found that endodontic disinfectants significantly reduced the adhesion of *E. faecalis* to dentin. Also, Kumar et al^13^ assessed the efficiency of laser activation of NaOCl in eliminating multispecies biofilms from the mesial root of permanent molars and found that the laser agitation resulted in effective isthmus cleaning. The present study evaluated the removal of mature *E. faecalis* laboratory biofilms in a human complex root canal system after Er,Cr:YSGG laser in PIPS was used to activate 5.25% NaOCl irrigation solution and the results obtained by AFM analysis of surface roughness. A conventional syringe and passive ultrasonic irrigation (PUI) procedures were used as a reference for comparison.

## Methods

 The Declaration of Helsinki was followed in this investigation. Ethical approval for the procedures of the present study was obtained (10-2022-488).

###  Specimen selection and preparation

 Seventy-five extracted mandibular first and second molars without root canal fillings, root caries, or restorations were obtained and cleaned immediately after extraction. All the samples were placed in a glass container that contained distilled water with 0.1% thymol crystals (Lab Grade, Lab Alley, Texas, USA) until the experiment day.

 A diamond disc (OSA-E28, Osakadent Group Ltd., Guangdong, China) was used for cutting off the tooth crowns, and the mesial roots of all molar teeth were separated from the rest of the teeth to achieve roots with a length of 12 mm from the apex. A #10 stainless-steel K-type hand file (Dentsply, Maillefer, Ballaigues, Switzerland) was used to locate the apical foramen. After sighting the file tip through the apical foramen, 1 mm was subtracted from the measured file length to determine the working length (WL). All the root canals were prepared to this WL up to #25/.04 NiTi rotary files (X3 Never Break Serious, EasyinSmile, New Jersey, USA) at speed and torque recommended by the manufacturer.

 During instrumentation, 1 mL of 5.25% NaOCl (Cerkamed, Stalowa Wola, Poland) was administered after each file size, using a 30-gauge irrigation needle with side vents (EndoTop, Hangzhou EndoTop Medi-Tech Co. Ltd., Zhejiang, China). 1 mL of 17% EDTA was used as the final irrigant (Cerkamed, Stalowa Wola, Poland), and the solution was left in situ for 3 minutes. The solution was activated with an ultrasonic device (Guilin Woodpecker Medical Instrument Co. Ltd, Guangxi, China) for 30 seconds. In the final stage of the process, all the root samples were irrigated with 5 mL of distilled water (Pioneer Company, Baghdad, Iraq) to eliminate the remaining irrigation solutions. The root canals and the outer surfaces of the teeth were dried with paper points (Sure-endo, Sure Dent Corporation, Gyeonggi-do, Korea) and externally with paper towels. All the roots were placed individually in Eppendorf tubes (LabServ, Thermo Fisher Scientific, Gurugram, India) upright and autoclaved for 20 minutes at 121 °C under 15 psi pressure.

###  Bacterial inoculation 

 Multiple *E. faecalis* colonies were taken from the agar plate (Himedia, Mumbai, India) and activated by placing them in brain-heart infusion (BHI) broth (Himedia, Mumbai, India) a day before. Then 1 mL of bacterial infusion was diluted by adding 8 mL of normal saline to obtain a suspension equal to the McFarland standard (1.5 × 10^8^ colony forming units CFU/mL). The bacterial suspension was injected into the cleaned root canals using a disposable syringe and a 30-gauge irrigation needle until they were filled (except for 15 roots which served as a negative control). Each root specimen was immersed in 1.5 mL of BHI broth after introducing the bacteria into the canals.All the sample tubes were kept at 37 °C in an aerobic warm environment for 30 days. Re-inoculations were carried out every three days, and a fresh BHI was used daily to ensure the presence of live bacteria during the incubation period. All the steps were performed in a sterile environment.

###  Treatment groups

 At the point of the incubation period termination, the liquid medium was drawn out from the tubes. The sixty samples were irrigated with 5 mL of distilled water and divided randomly into four groups: (A) The positive control group did not undergo any therapy (n = 15); (B) The samples were irrigated with 5.25% NaOCl delivered by an irrigation needle (n = 15); (C) Passive ultrasonic activation of 5.25% NaOCl (n = 15); and (D) Er,Cr:YSGG laser at 60 µs/pulse, 5 Hz, 0.25, 0.5, 0.75, 1, and 1.25 W using MZ6 6-mm length tip in PIPS protocol agitation of 5.25% NaOCl (n = 15).

 Each sample of the last three groups was subjected to several procedures: all sample surfaces were cleaned with sterilized cotton pellets immersed in 5.25% NaOCl and then mounted in plastic tubes filled with alginate impression material (Kromalgin, Vannini Dental, Grassina, Italy) for easy handling of the samples. For group B, a 30-gauge irrigation needle was used to irrigate the samples with 5.25% NaOCl, which was left within the canals for 2 min and then washed with 5 mL of distilled water. Group C samples were also irrigated with 5.25% NaOCl, which was left within the canals for 2 minutes, activated by a passive ultrasonic tip for 60 seconds, and washed with 5 mL of distilled water. The device tip was placed 1 mm shorter than the estimated WL.

**Figure 1:**
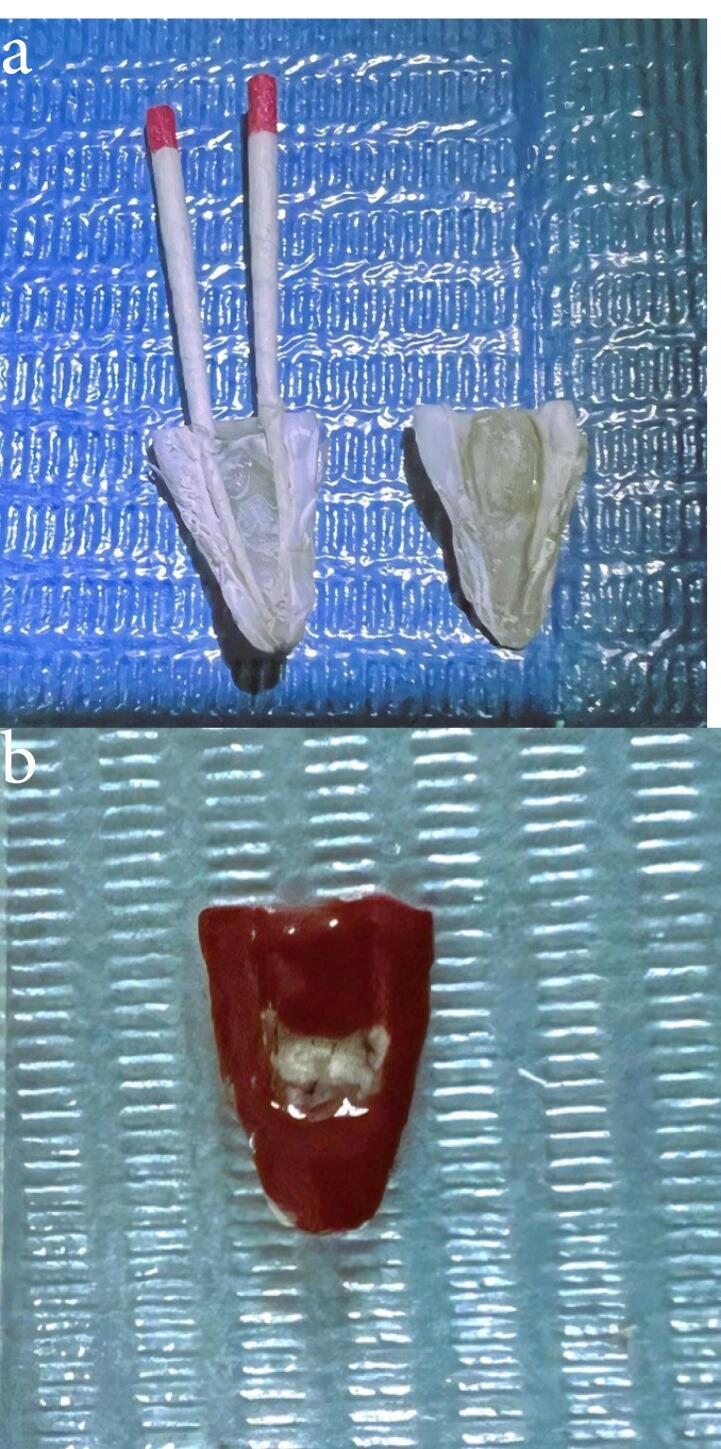
Sectioning of the root for AFM examination (a) Root sample after longitudinally divided (b) The middle area of the isthmus is located on one root section prepared for the AFM test

**Figure 2:**
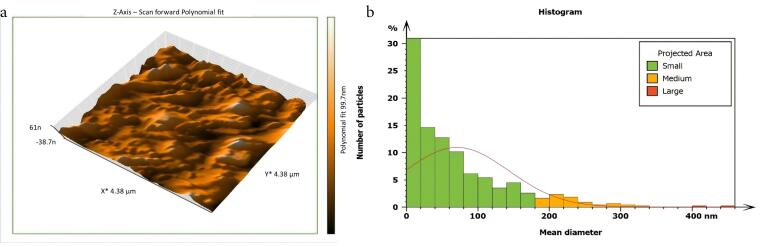
AFM imaging of not inoculated isthmus surface 6 mm from the apex (a) Three dimensions (b) The histogram of mean diameter

 Group D samples were irrigated with 5.25% NaOCl, which was left inside the canals for 2 minutes. Throughout this time, the fluid was activated by 2780 nm Er,Cr:YSGG laser irradiation (Waterlase iPlus, Biolase Inc., CA, USA) for 60 seconds with a change of power value for each sample. Infrared laser safety glasses (Innovative Optics, Hemlock Lane North Maple Grove, USA) were worn before laser activation. A newly designed water Lase iPlus/MD glass tip was used with a tip diameter of 600 μm and a length of 6 mm (MZ6 Zip, Biolase Inc., CA, USA). The laser unit’s water and air spray were both set to “off.” During laser application, the tip was placed just in the canal orifice, remained stationary, and did not move apically into the root canal. Laser operation proceeded for 30 seconds of “on” time, followed by 30 seconds of “off” time, and this sequence was repeated twice (for a total of 60 seconds of activation). As explained in the literature, the resting period between laser agitation cycles allowed for the most active forms of NaOCl release. After that, the canal was irrigated with a sterile saline solution for 60 seconds. Properly fitting paper point cones were placed in the root canals to prevent tooth fragments from entering the endodontic canals and the isthmus area. All the roots were grooved longitudinally on their outer surface using a diamond disc, and a chisel was used to cut specimens into halves (Figure 1a). A middle area of the isthmus was marked for examination by AFM (Nanosurf, Liestal, Switzerland) (Figure 2b).

###  Statistical analysis

 The data were analyzed using SPSS 21 (IBM, Armonk, New York, USA). The data were reported as means, while categorical data were displayed using standard deviations. ANOVA was used to compare the means of the tests. Data were expressed as mean ± SD. The LSD test was used to compute the significant differences among the tested means, and the letters (A, B, C, D, and E) for rows showed the levels of significance, highly significant beginning with the letter (A) and reducing with the last one. Similar letters mean no significant differences between the tested means. *P* ≤ 0.05 was considered a significant value.

## Results

**Figure 3:**
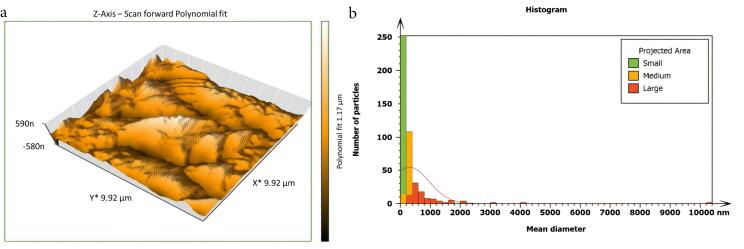
AFM imaging of inoculated, untreated isthmus surface about 6 mm from the apex (a) Three dimensions (b) The histogram of the particle’s mean diameter

**Figure 4:**
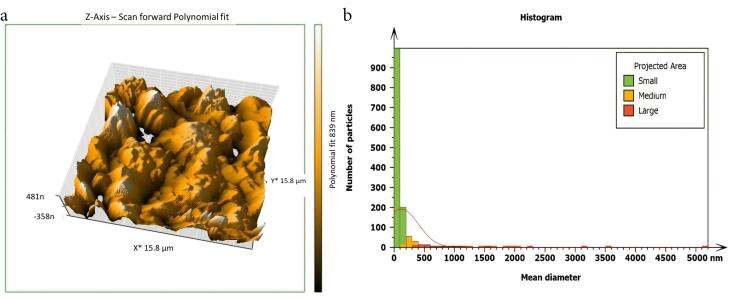
AFM imaging of the middle isthmus surface after being treated with 5.25% NaOCl + SI (a) Three dimensions (b) The histogram of mean diameter

**Figure 5:**
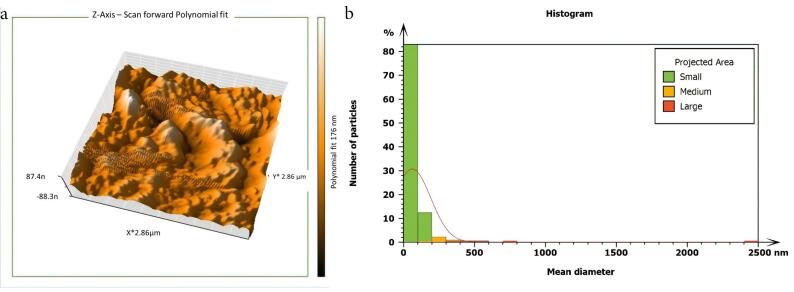
AFM imaging of the middle isthmus surface after being treated with 5.25% NaOCl & passive ultrasonic activation. (a) Three dimensions (b) The histogram of mean diameter

**Figure 6:**
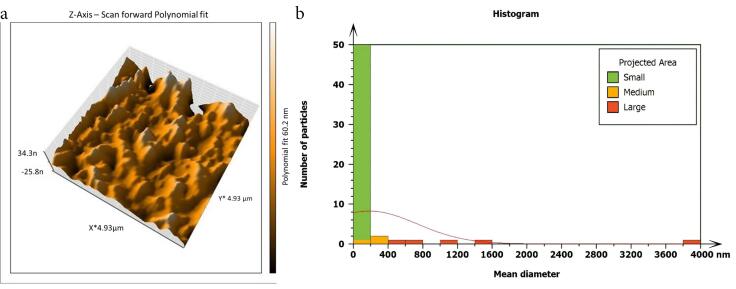
AFM imaging of the isthmus surface after beig treated with 5.25% NaOCl agitated with Er, Cr: YSGG laser in PIPS (60 µs/pulse at 5 Hz, 1 W) (a) Three dimensions. (b) The histogram of mean diameter

**Figure 7:**
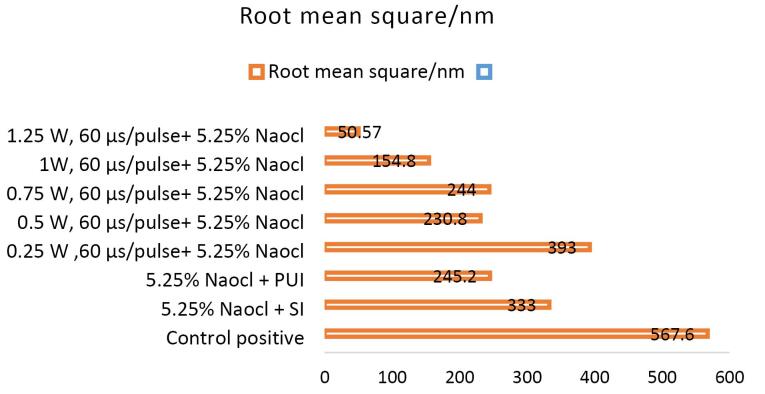
Bar charts showing the root mean square of positive control& other test groups

 Non-inoculated isthmus area was illustrated in a three-dimensional image (Figure 2a), in addition to a histogram that showed the dominance of small particles with a low mean diameter value = 52.82 nm (Figure 2b). Figure 3 shows the 3D images of the non-treated middle isthmus surface. Also, a histogram of the mean diameter displayed small particles with a high mean diameter value = 76.24 nm. Figure 4 shows the isthmus surface after being treated with 5.25% NaOCl by irrigation syringe method without any agitation. In addition, the mean diameter of small surface particles, about 52.74 nm, is displayed in the histogram (Figure 4b). Figure 5 shows the isthmus of treated roots with NaOCl agitated with PUI. Figure 6 shows the images of the isthmus surface treated with 5.25% NaOCl and agitated with an Er,Cr:YSGG laser (at 60 µs/pulse, 5 Hz, and 1.25 W), along with a histogram that displays a low value for small particles mean diameter of about 42.02 nm. Values of the root mean square of positive control and other test groups after statistical analysis are presented in Table 1 and a statistical bar chart (Figure 7). Bacterial biofilms decreased significantly in all the test groups (*P* < 0.05). Er,Cr:YSGG laser in PIPS (at 60 µs/pulse, 5 Hz, 1.25 W) agitation of 5.25% NaOCl results were highly significant compared to the other test groups’ results followed by the PUI group.

**Table 1:** The root mean square parameter’s means and standard deviations in the positive control and other test groups; means were compared by two-way ANOVA

**Tested groups of 5.25% NaOCl**	**Sq/nm** **Mean±SD**	***P***** value**
Negative control (n = 15)	17.73 ± 2.9	0.001
Positive control (n = 15)	E 567.6 ± 21.4	
5.25% NaOCl + SI (n = 15)	D 333.0 ± 22.1	0.01
5.25% NaOCl + PUI (n = 15)	C 245.2 ± 21	
Laser groups + 5.25% NaOCl (n = 15)		
0.25 W, 60 µs/pulse + 5.25% NaOCl	E 393.0 ± 10	0.001
0.5 W, 60 µs/pulse + 5.25% NaOCl	C 230.8 ± 39.9	
0.75 W, 60 µs/pulse + 5.25% NaOCl	C 244.0 ± 16	
1W, 60 µs/pulse + 5.25% NaOCl	B 154.8 ± 13.4	
1.25 W, 60 µs/pulse + 5.25% NaOCl	A 50.57 ± 4.2	
*P *value	0.001	

LSD test was used to calculate significant differences between the tested means; the letters (A, B, C, D, and E) represent the levels of significance, highly significant start from the letter (A) and decreasing with the last one. Similar letters mean no significant differences between the tested mean. Sq: Root mean square (a roughness parameter); nm: nanometer (unit of length); SI: Syringe irrigation; LSD: The least significant difference; PUI: Passive ultrasonic irrigation.

**Figure 8:**
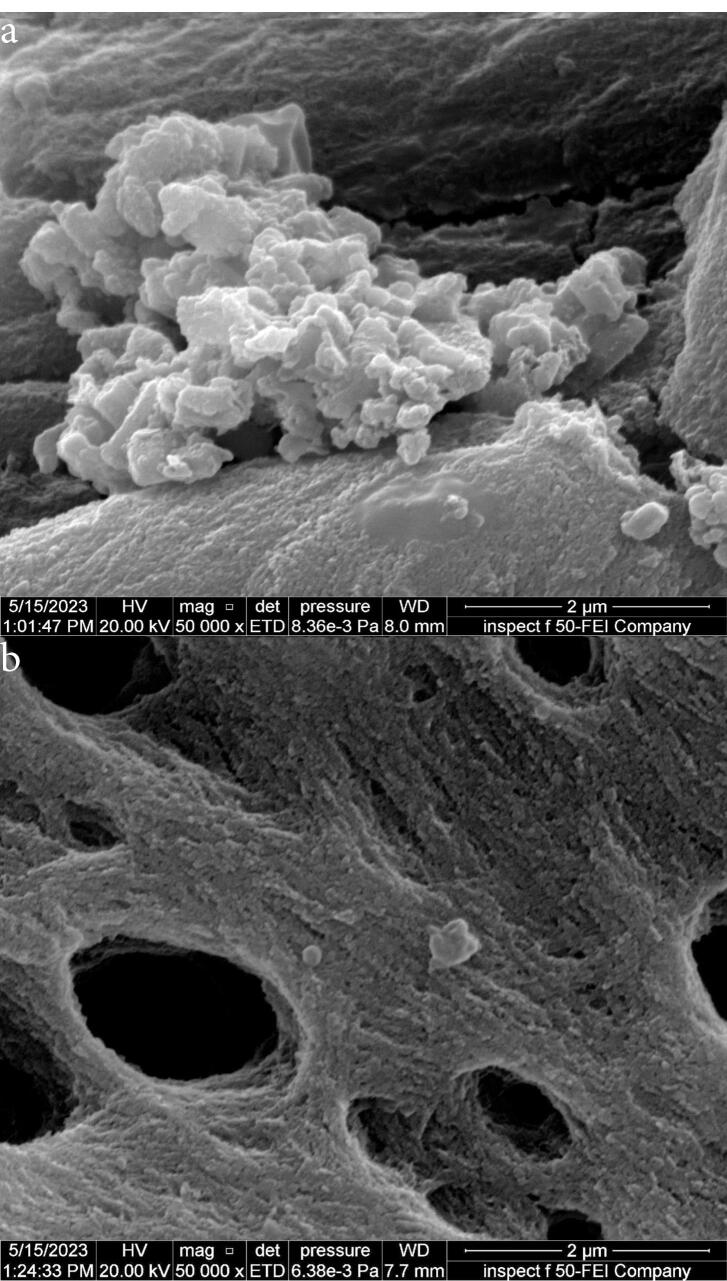
FE-SEM (50 000x) magnification of the isthmus area about 6 mm from the apex (a) Positive control sample (b) The same area after being treated with 5.25% NaOCl + Er, Cr: YSGG laser using PIPS

Figure 8 shows images from field emission scanning electron microscopy (FE-SEM) taken close to 6 mm from the apex of the isthmus before and after root canals irrigation using 5.25% NaOCl agitated with Er,Cr:YSGG laser at 60 µs/pulse, 5 Hz, and 1.25 W. Figure 8 shows a mature biofilm covering the isthmus surface, while after laser PIPS agitation, a clean surface was achieved with isolated smashed bacterial cells (Figure 8b).

## Discussion

 Endodontic therapy’s main goal is to clean the root canal completely; however, this task is challenging due to the complicated structure of the canal system. Simple gram-positive microorganisms dominate the microbial community of teeth with persisting apical periodontitis. *E. faecalis* was selected for this investigation because it is thought to be the most resistant microbe detected in infected root canals, having particular mechanisms in constructing a biofilm, significant virulence-related factors, adhesion capacity to dentin collagen, survivability in harsh circumstances, capability to resist root canal treatment, and easy to grow in vitro.^14^ Past experiments have used various materials to produce bacterial biofilms, such as nitrocellulose membrane filters,^15^ hydroxyapatite discs, or previously extracted teeth.^16^ In this study, anatomically complicated extracted human teeth were inoculated with *E. faecalis* in a laboratory environment. The time required for biofilm development differs in research (15 minutes to 60 days).^17^ A prolonged period of incubation results in more mature biofilms. In the current work, infected teeth underwent incubation at 37 °C for 30 days to allow *E. faecalis* biofilms to mature. NaOCl was selected in the present study due to its ability to dissolve vital and necrotic pulp tissues, high alkalinity, and powerful antimicrobial action. The effectiveness of irrigation is influenced by irrigation quantity, vicinity to the apex, solution temperature, and the dynamics of fluids produced by activation methods.^18^

 In past experiments, both culture procedures and molecular methods have been used to determine the number of viable bacteria in the root canals and dentinal tubules.^16^ Also, a confocal laser microscope (CLSM) can detect the viability of bacteria colonizing the root canal wall, lateral canal, and isthmus.^13^ This study used an AFM to analyze the samples. It is easy to apply, precise, available, and more cost-effective than CLSM. In 2015, López-Jiménez et al^19^ used AFM to visualize the alterations induced on *E. faecalis* surface after treatment with Er,Cr:YSGG and diode lasers. They found that AFM is a good tool and could constitute a measure of antimicrobial effect by determining microbial viability.

 AFM is used as a supplementary tool for investigating antibacterial mechanisms by exposing the change in the roughness of the surface inoculated with *E. faecalis* biofilm. The topography of the surface of the isthmus was studied, and measurements of surface roughness, particle size, and particle analysis were taken. Surface roughness was determined by computing the parameters of the surface profile: root mean square (Sq), average roughness (Sa), and maximum height (Sz). The roughness parameters are determined by analyzing topographical scans of the sample’s isthmus surface. In this study, Sq depended on the root square of the surface height distribution and was more responsive to significant deviations from the mean line than average roughness.^10^ Based on the results, the calculated root mean square value of the isthmus surface that was uncultivated with bacteria was extremely low (approximately 17.73 ± 2.9 nm), while the Sq of the surface after the development of mature biofilm was significantly high (around 567.6 ± 21.4 nm). Also, the mean diameter value of the small particles of the cultivated isthmus was higher (76.24 nm). The increase in roughness of the surface suggests that the biofilm formation led to a more textured and uneven surface. Additionally, the higher mean diameter value of small particles indicates that the presence of bacterial cells on the surface caused an accumulation of material, resulting in larger particle sizes. The external isthmus surface treated with 5.25% NaOCl activated by Er,Cr:YSGG laser displayed in a 3D AFM image contained fewer high peaks and troughs and a random or low bumpy surface. The Sq value was about 50.57 ± 4.2 nm, which is significantly less than the values measured after other methods (SI and PUI), indicating decreased *E. faecalis* biofilm on the isthmus surface by laser. PIPS was more effective than traditional methods. Also, the mean diameter of small particles was 42.02 nm, which is noticeably less than that of surface particles treated with a syringe irrigation protocol (52.74 nm).

 The external isthmus surface featured a few noticeable peaks and low troughs with a random or low bumpy surface after being treated with 5.25% NaOCl activated by a passive ultrasonic system. The Sq value of about 245.2 ± 21 nm was less than the value measured after the syringe irrigation method (333.0 ± 22.1 nm), which means that the treatment mechanism by passive ultrasonic agitation of NaOCl was more effective than the conventional method; the reduction in the roughness of the surface reflected this result. Also, the particle mean diameter of small particles was about 42.06 nm, less than the mean diameter of the surface treated with syringe irrigation alone. PUI and PIPS were significantly more effective against bacterial biofilms than syringe irrigation, which might be attributed to the acoustic microstreaming generated by these mechanisms.^20,21^ The second factor is cavitation, which weakens the cell membrane and makes it easier for irrigants to penetrate bacterial cells,^22^ maybe resulting in better clearance of biofilms from areas that are hard to reach.

 Generally, the most significant surfaces were 567.6 ± 21.4 and 333.0 ± 22.1 nm in the positive control and the syringe irrigation groups, respectively. The lowest surface was 50.57 ± 4.2 nm inoculated with *E. faecalis* biofilm treated with 5.25% NaOCl agitated by Er,Cr:YSGG laser in PIPS at 1.25 W and 60 µs pulse duration. The extremely short pulse durations (60 µs) generate higher peak power than the lengthier ones; this generates strong pressure and shockwaves that propagate three-dimensionally within the root canals filled with fluids, eliminating the need to put the tip near the morphologically thinning apical third.^23^ These findings revealed that Er,Cr:YSGG laser agitation of NaOCl irrigation solution is more effective than irrigation agents that are not agitated or agitated by a passive ultrasonic device.

 Many studies have tested the antimicrobial effect of Er,Cr:YSGG laser and other lasers and have obtained similar results but at different laser settings and different root canal systems. Schoop et al^24^ employed Er,Cr:YSGG laser at 1.5 and 2.5 W powers with no air or water and found that laser agitation significantly reduced intracanal bacterial counts compared to a control group. Golob et al^25^ tested the PIPS approach’s effectiveness in reducing *E. faecalis* in root canals using a lower energy level and various NaOCl concentrations. They discovered that laser agitation using PIPS with 5% NaOCl effectively eliminated the bacterial biofilm. Betancourt et al^26^ used a fabricated root canal model to compare the antimicrobial effectiveness of PUI and Er,Cr:YSGG laser-agitated NaOCl upon *E. faecalis* biofilm and injuries on bacterial structures by AFM. They discovered that laser activation enhanced the bactericidal effectiveness of 0.5% NaOCl.

 Lastly, this investigation was constrained by the formation of microscopic fragments during the cutting stage, which could increase the surface roughness value. However, this drawback was minimized using a paper point placed in the canal to prevent debris from entering the isthmus during the cutting phase. Another drawback that might have affected the results is the anatomic variations of the isthmus areas of the selected teeth.

## Conclusion

 Under the conditions of this study and according to AFM analysis, it is possible to conclude that Er,Cr:YSGG laser and ultrasonic agitation of NaOCl provide greater biofilm removal than typical irrigation methods by an irrigation syringe without any activation. After testing many laser powers, it can be deduced that Er,Cr:YSGG laser in PIPS at 60 µs/pulse, 5 Hz, and 1.25 W was the best method to improve the antimicrobial effectiveness of 5.25% NaOCl against 30-day-old *E. faecalis* biofilms in the difficult-to-reach isthmus region.

## Competing Interests

 No conflicts of interest.

## Ethical Approval

 The Research and Ethics Committee of the Institute of Laser for Postgraduate Studies, Baghdad University, approved the study (Date: 19/10/2022, Number # 488).

## References

[1] Betancourt P, Sierra JM, Camps-Font O, Arnabat-Domínguez J, Viñas M (2019). Er,Cr:YSGG laser-activation enhances antimicrobial and antibiofilm action of low concentrations of sodium hypochlorite in root canals. Antibiotics (Basel).

[2] Robberecht L, Delattre J, Meire M (2023). Isthmus morphology influences debridement efficacy of activated irrigation: a laboratory study involving biofilm mimicking hydrogel removal and high-speed imaging. Int Endod J.

[3] Rosen E, Tsesis I, Elbahary S, Storzi N, Kolodkin-Gal I (2016). Eradication of Enterococcus faecalis biofilms on human dentin. Front Microbiol.

[4] Cullen JK, Wealleans JA, Kirkpatrick TC, Yaccino JM (2015). The effect of 825% sodium hypochlorite on dental pulp dissolution and dentin flexural strength and modulus. J Endod.

[5] Al-Karadaghi TS, Franzen R, Jawad HA, Gutknecht N (2015). Investigations of radicular dentin permeability and ultrastructural changes after irradiation with Er,Cr:YSGG laser and dual wavelength (2780 and 940 nm) laser. Lasers Med Sci.

[6] Jassim AK, Jawad HA (2022). Cavity disinfection using Er,Cr:YSGG laser induced photoacoustic streaming technique. Iraqi J Laser.

[7] Al-Karadaghi TS, Gutknecht N, Jawad HA, Vanweersch L, Franzen R (2015). Evaluation of temperature elevation during root canal treatment with dual wavelength laser: 2780 nm Er,Cr:YSGG and 940 nm diode. Photomed Laser Surg.

[8] Rasheed SS, Jawad HA (2021). Smear layer removal from the apical third using the Er,Cr:YSGG photon-induced photoacoustic streaming. Iran Endod J.

[9] Muhammed FS, Jawad HA (2021). Pulsed Er,Cr:YSGG laser for surface modification of dental zerconia ceramic. Iraqi J Laser.

[10] Kumar BR, Rao TS (2012). AFM studies on surface morphology, topography and texture of nanostructured zinc aluminum oxide thin films. Dig J Nanomater Biostruct.

[11] Gadegaard N (2006). Atomic force microscopy in biology: technology and techniques. Biotech Histochem.

[12] Kishen A, Sum CP, Mathew S, Lim CT (2008). Influence of irrigation regimens on the adherence of Enterococcus faecalis to root canal dentin. J Endod.

[13] Kumar K, Teoh YY, Walsh LJ (2023). Root canal cleaning in roots with complex canals using agitated irrigation fluids. Aust Endod J.

[14] Al-Shawi DA, Al-Quraishi G (2023). Multidrug resistant Enterococcus faecalis isolated from root canals and its relationship with the presence of some virulence genes. Egypt J Hosp Med.

[15] Ghivari SB, Bhattacharya H, Bhat KG, Pujar MA (2017). Antimicrobial activity of root canal irrigants against biofilm forming pathogens- an in vitro study. J Conserv Dent.

[16] Hoedke D, Kaulika N, Dommisch H, Schlafer S, Shemesh H, Bitter K (2021). Reduction of dual-species biofilm after sonic- or ultrasonic-activated irrigation protocols: a laboratory study. Int Endod J.

[17] Cheng X, Tian T, Tian Y, Xiang D, Qiu J, Liu X (2017). Erbium:yttrium aluminum garnet laser-activated sodium hypochlorite irrigation: a promising procedure for minimally invasive endodontics. Photomed Laser Surg.

[18] George R (2019). Evaluation of the evidence of effectiveness of ultrasonic activated irrigation for root canal treatment. Evid Based Dent.

[19] López-Jiménez L, Arnabat-Domínguez J, Viñas M, Vinuesa T (2015). Atomic force microscopy visualization of injuries in Enterococcus faecalis surface caused by Er,Cr:YSGG and diode lasers. Med Oral Patol Oral Cir Bucal.

[20] Olivi G, DiVito EE. Advanced laser-activated irrigation: PIPSTM technique and clinical protocols. In: Olivi G, De Moor R, DiVito E, eds. Lasers in Endodontics: Scientific Background and Clinical Applications. Cham: Springer; 2016. p. 219-91. 10.1007/978-3-319-19327-4_11.

[21] Rasheed SS, Jawad HA (2021). Permeability of radicular dentine after using different irrigant activation techniques including photo induce photoacoustic streaming technique. Iraqi J Laser.

[22] Mahfouze AL, El Gendy AA, Elsewify TM (2020). Bacterial reduction of mature Enterococcus faecalis biofilm by different irrigants and activation techniques using confocal laser scanning microscopy An in vitro study. Saudi Endod J.

[23] Olivi G (2013). Laser use in endodontics: evolution from direct laser irradiation to laser-activated irrigation. J Laser Dent.

[24] Schoop U, Goharkhay K, Klimscha J, Zagler M, Wernisch J, Georgopoulos A (2007). The use of the erbium, chromium:yttrium-scandium-gallium-garnet laser in endodontic treatment: the results of an in vitro study. J Am Dent Assoc.

[25] Golob BS, Olivi G, Vrabec M, El Feghali R, Parker S, Benedicenti S (2017). Efficacy of photon-induced photoacoustic streaming in the reduction of Enterococcus faecalis within the root canal: different settings and different sodium hypochlorite concentrations. J Endod.

[26] Betancourt P, Merlos A, Sierra JM, Camps-Font O, Arnabat-Dominguez J, Viñas M (2019). Effectiveness of low concentration of sodium hypochlorite activated by Er,Cr:YSGG laser against Enterococcus faecalis biofilm. Lasers Med Sci.

